# The Intersection of Rural Residence and Minority Race/Ethnicity in Cancer Disparities in the United States

**DOI:** 10.3390/ijerph18041384

**Published:** 2021-02-03

**Authors:** Whitney E. Zahnd, Cathryn Murphy, Marie Knoll, Gabriel A. Benavidez, Kelsey R. Day, Radhika Ranganathan, Parthenia Luke, Anja Zgodic, Kewei Shi, Melinda A. Merrell, Elizabeth L. Crouch, Heather M. Brandt, Jan M. Eberth

**Affiliations:** 1 Rural & Minority Health Research Center, Arnold School of Public Health, University of South Carolina, Columbia, SC 29210, USA; cathrynm@email.sc.edu (C.M.); BENAVIDG@email.sc.edu (G.A.B.); radhika@email.sc.edu (R.R.); azgodic@email.sc.edu (A.Z.); kewei.shi@yale.edu (K.S.); mmerrell@mailbox.sc.edu (M.A.M.); crouchel@mailbox.sc.edu (E.L.C.); jmeberth@mailbox.sc.edu (J.M.E.); 2 Big Data Health Science Center, Arnold School of Public Health, University of South Carolina, Columbia, SC 29208, USA; 3 College of Arts and Sciences, University of South Carolina, Columbia, SC 29208, USA; 4 Department of Epidemiology & Biostatistics, Arnold School of Public Health, University of South Carolina, Columbia, SC 29208, USA; mknoll@email.sc.edu; 5 Department of Exercise Science, Arnold School of Public Health, University of South Carolina, Columbia, SC 29208, USA; Krday@email.sc.edu; 6 College of Social Work, University of South Carolina, Columbia, SC 29208, USA; lukep@email.sc.edu; 7 Department of Health Services Policy and Management, Arnold School of Public Health, University of South Carolina, Columbia, SC 29208, USA; 8 St. Jude Children’s Research Hospital, Memphis, TN 38105, USA; heather.brandt@stjude.org

**Keywords:** rural, racial/ethnic minorities, cancer disparities, access to care, social determinants of health, cancer surveillance, cancer outcomes

## Abstract

One in every twenty-five persons in America is a racial/ethnic minority who lives in a rural area. Our objective was to summarize how racism and, subsequently, the social determinants of health disproportionately affect rural racial/ethnic minority populations, provide a review of the cancer disparities experienced by rural racial/ethnic minority groups, and recommend policy, research, and intervention approaches to reduce these disparities. We found that rural Black and American Indian/Alaska Native populations experience greater poverty and lack of access to care, which expose them to greater risk of developing cancer and experiencing poorer cancer outcomes in treatment and ultimately survival. There is a critical need for additional research to understand the disparities experienced by all rural racial/ethnic minority populations. We propose that policies aim to increase access to care and healthcare resources for these communities. Further, that observational and interventional research should more effectively address the intersections of rurality and race/ethnicity through reduced structural and interpersonal biases in cancer care, increased data access, more research on newer cancer screening and treatment modalities, and continued intervention and implementation research to understand how evidence-based practices can most effectively reduce disparities among these populations.

## 1. Introduction

Rural populations in the United States comprise as much as 20% of the total population (i.e., as many as 59 million persons) [1]. Defining “rural” is objectively and subjectively challenging, as it is often driven by administrative geographic units (e.g., counties) for which there may be considerable variation in geographic and population size [1]. Regardless of the formal definition of “rural”, a “rural” area is typically characterized by small population density or size and notable distance to a metropolitan/urban area [1]. Three in four rural persons in the United States live in the South or Midwest. However, the most isolated rural areas of the country are in the West, and even the population-dense Northeast includes sizable pockets of rural populations. Rural populations face a myriad of structural, socioeconomic, economic, environmental, and access-to-care barriers that put them at greater risk for higher prevalence of poor health behaviors (e.g., smoking, sedentary behavior), lower utilization of cancer-relevant preventive services, lower odds of receiving guideline-concordant treatment, and poorer cancer outcomes from incidence to survivorship [2,3,4,5,6,7,8].

Definitions of race and ethnicity are also socially and administratively driven. In this article, we categorize racial/ethnic groups as non-Hispanic White (the majority group), non-Hispanic Black, Hispanic, American Indian/Alaska Native, Asian, Native Hawaiian, and Other Pacific Islander. These are racial/ethnic groupings used by the Office of Management and Budget and the National Institute on Minority Health and Health Disparities [9,10]. Tangibly speaking, race and ethnicity are individual-level indicators of structural, institutional, and/or interpersonal disadvantage (or, conversely, advantage) that systematically and differentially affect population subgroups. Racial/ethnic minority groups are affected by both unique and similar historical and contemporary racism (both interpersonal and systemic) and other structural barriers to optimal health. Non-Hispanic Black populations have been affected by centuries of enslavement and brutality and have been particularly affected by segregationist policies (e.g., Jim Crow laws, redlining, exclusionary zoning) that have long-lasting social and health impacts beyond political, judicial, and legislative remedies (e.g., reconstruction, the Brown vs. the Board of Education decision, and the Fair Housing Act of 1968) [11,12]. American Indian and Alaska Native populations have similarly experienced sustained ill-health effects due to colonialism, forced migration, and racist policies (e.g., forced sterilization, delayed voting rights) [11]. Hispanic populations, comprising the largest current and fastest-growing rural population in the U.S., have been particularly affected by restrictive and xenophobic immigration policies that may affect health and access to healthcare services regardless of immigration status [12,13,14]. Asian and Native Hawaiian and Other Pacific Islander populations have been affected by discriminatory legislation and policies (e.g., exploitation of Chinese laborers in the late 19th century, exclusionary immigration policies, internment of Japanese Americans in World War II) [15,16]. These historic traumas, in addition to continued discriminatory policies and interpersonal biases, have limited minority populations’ access to healthcare services, put them at greater odds of engaging in poorer health behaviors or being exposed to environmental carcinogens, make them less likely to receive optimal cancer treatment, and contribute to their greater cancer burden across the continuum [17,18,19,20,21,22,23].

**Figure 1:**
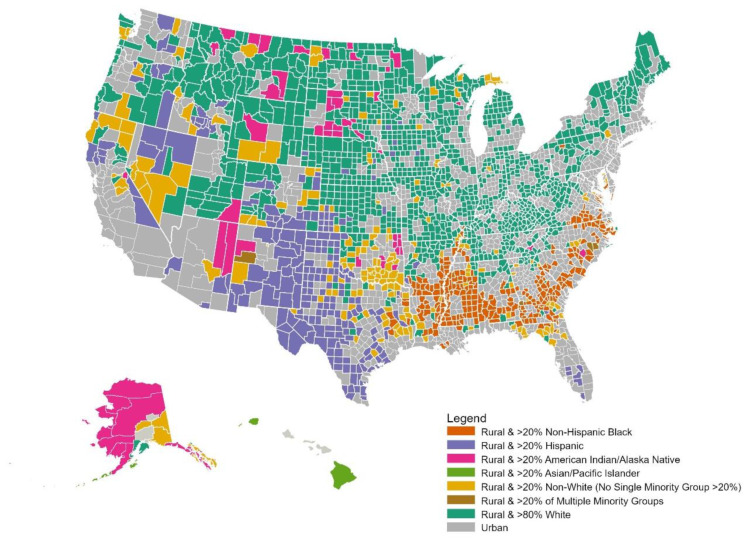
Racial/ethnic composition across rural counties, 2018. Rural and urban counties are defined by the non-metro and metro designations from the United States Department of Agriculture [30]. Racial/ethnic composition by county is based on the 2018 Census Bureau Population Estimates Program [31].

The intersection of rurality and minority race/ethnicity in the United States is critical to our understanding of health disparities broadly, and cancer disparities specifically, but is often overlooked [24,25,26,27]. Overall, one in every 25 people in the U.S. is a non-White rural person (i.e., 22% of the U.S. rural population) [28]. Although most rural persons are White, 9% and 8% belong are Hispanic and Black, respectively. Rural, non-Hispanic Black and Hispanic populations reside primarily in the South (Figure 1), whereas rural American Indian/Alaska Native populations primarily reside in Alaska, the northern plains, and the Southwest. Although rural populations overall have declined because of outmigration and urban sprawl, rural Hispanic populations have grown in recent decades [29]. Furthermore, there are many counties throughout the country in which notable proportions of the populations are non-White (i.e., smaller proportions of racial/ethnic minority populations combine for a notable proportion of the total population). The intersection of the aforementioned structural barriers experienced by rural and racial/ethnic minority populations will be the focus of this paper. We begin our review by describing structural and contextual factors that disproportionately affect rural racial/ethnic minority populations and contribute to cancer disparities. Next, we summarize the literature from the past 20 years on disparities experienced by rural, racial/ethnic minority populations across the cancer control continuum. Finally, we discuss the opportunities for observational and interventional research to mitigate these disparities.

## 2. A Conceptual Framework

**Figure 2:**
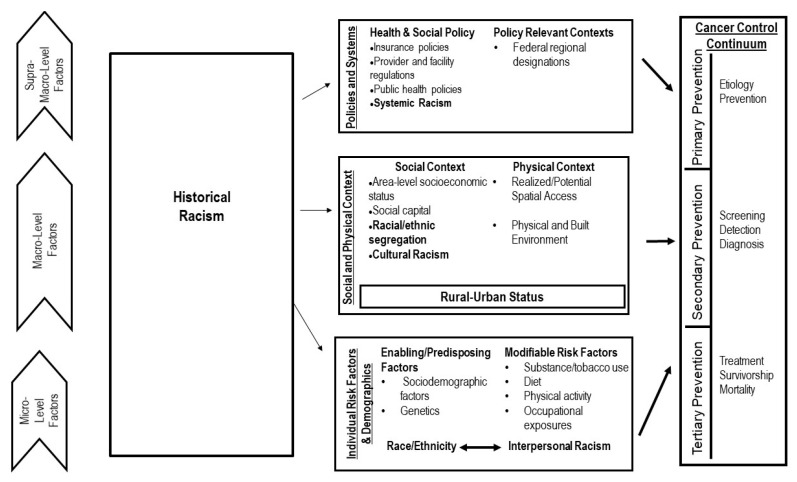
A conceptual framework of the multilevel influence of racism on rural cancer disparities across the continuum. The role of racism across levels in noted in bold.

Structural, social, and environmental factors at the macro and supramacro levels (e.g., policies, area-level poverty, area-level distance to care), in addition to micro-level factors (e.g., insurance status, age, behaviors), affect cancer across the continuum from etiology to survivorship. Supramacro factors, such as state-level policies, may have both an effect on lower level macro and micro factors and, subsequently, cancer outcomes across the continuum. For example, state-level Medicaid expansion, which is less common in largely rural states with higher proportions of Black residents, may affect the macro-level factor of potential access to care, the micro-level factor of individual insurance status and, subsequently, may affect cancer screening uptake, cancer staging, and cancer treatment [32,33,34]. To better contextualize the interplay of race/ethnicity and rurality, here we also adapt a framework from our previous work to depict how the intersection of rurality and race/ethnicity, combined with these multilevel factors, affects cancer outcomes across the continuum and demonstrate how racism at different levels may affect cancer outcomes by drawing from Gee and Ford, as well as Williams (Figure 2) [35,36,37]. Our adapted framework notes that historical racism (also referred to as “intergenerational drag”, as characterized by Gee and Ford) can affect different levels—the micro/individual level (interpersonal racism), the macro-level (racial/ethnic segregation, cultural racism), and the supramacro level (systemic racism)—also noting that racism has effects between levels as well. We place systemic racism and segregation at different levels, as often higher-level factors (e.g., state, federal policies that are systemic) have the greatest impact and/or are more effectively quantitatively measured at lower levels (e.g., county- or neighborhood-level residential segregation). The cancer continuum can be considered discretely within primary, secondary, or tertiary prevention based on how public health and healthcare systems can intervene to address each area. As in our previous work, we here integrate both the National Cancer Institute’s Cancer Control Continuum and Wingo’s Framework for Cancer Surveillance to characterize rural cancer disparities within this continuum [37,38]. Areas of primary prevention include etiology and prevention, secondary prevention includes screening, early detection, and diagnosis, and tertiary prevention includes treatment, survivorship, and mortality. 

## 3. Social Determinants of Health and Their Role in Rural and Racial/Ethnic Disparities in Cancer

Rural racial/ethnic minority populations often fare worse than their non-minority rural and urban minority counterparts with regard to the social determinants of health and health outcomes, including cancer outcomes [39,40,41]. Here, we summarize how several social and structural determinants of health adversely affect rural racial/ethnic minority populations: structural racism, residential segregation, access to healthcare services, socioeconomic status, educational attainment, infrastructure, and environmental exposures.

### 3.1. Structural Racism and Residential Segregation

Interpersonal discrimination is recognized as an important social determinant of health that affects cancer outcomes [42,43]. However, both historical and systemic racism, such as residential segregation, also play a major role in observed racial and ethnic geographic health disparities [44,45]. Studies have shown that women, particularly Black women, born in the era of Jim Crow were more likely to be diagnosed with the breast cancer subtypes that have poorer prognoses [46]. Jim Crow laws were state and local regulations commonly found in the South that codified racial segregation in transportation, employment, restaurants, and other settings [46]. Similarly, in urban areas, living in a historically “redlined” area is associated with later stage at diagnosis and poorer cancer survival [47]. “Redlining” was another form of government-sponsored segregation that essentially prevented individuals in Black communities from successfully obtaining mortgages [47]. Segregation contributes to health disparities because it “isolates a minority group from amenities, opportunities, and resources that affect social and economic well-being” [48]. One qualitative study that focused on Black residents of the rural Deep South, for example, identified several determinants of health at the structural level, including lack of opportunity for physical activities; lack of access to healthy foods; cronyism and nepotism in workplaces that favor White residents, a determinant of health given the association between income and health; persistent stress from poverty and institutional racism, a physiological cause of health risk [49]. Not only are Black people who live in segregated areas more likely to be diagnosed with breast and lung cancer at a later stage, but racially isolated areas also have less access to diagnostic cancer technologies [21,50]. Prior studies have also revealed that structural racism is harmful to residents of segregated areas regardless of race. Both Black and White breast cancer patients living in areas with high levels of Black segregation regardless of area-level poverty were less likely to receive appropriate care, and cancer survivors in similar areas had lower health-related quality of life even after accounting for area-level poverty, suggesting a systemic problem [51,52]. Although the intersection of race and economic class is important to consider, a national study found lung cancer mortality to be the highest among Black people living in the most segregated areas of the country, regardless of socioeconomic status or health insurance [21,53]. However, a study by Moss and colleagues found that screening rates were higher in more segregated areas regardless of race/ethnicity [54]. The intersection of residential segregation and rurality, as well as their compounding effects on health outcomes, is a critical area for additional research, especially as most rural minority populations live in the U.S. South, where the long-term effects of racist policies may be more prevalent, and interpersonal racism remains common [55].

### 3.2. Access to Healthcare Services

Healthcare access is often defined along five specific dimensions: availability, accessibility, accommodation, affordability, and acceptability [56]. Historical unethical treatment of racial/ethnic minority populations within the U.S. healthcare system (e.g., medical experimentation, forced sterilization, denial of needed medical treatment), as well as recent changes in rural healthcare systems, undermines access to healthcare [42]. Policies that segregated care among Black and White patients are embedded within the healthcare policy of the last century. The Hill-Burton Act passed in 1946 provided millions of dollars to increase the healthcare and public health infrastructure throughout the country but also allowed for racial segregation healthcare facilities [57]. Legal challenges and the Civil Rights Act led to the desegregation of hospitals. However, this also led to Black hospitals ultimately becoming financially inviable. This, coupled with cost slashing federal policies and changes to hospital payment structures in the 1970s and 1980s, led to a surge of hospital closures, particularly in the rural South [58]. In the context of both historical and contemporary policies, it is critical to examine these dimensions through the lenses of rurality, race/ethnicity, and cancer disparities.

Availability, accessibility, and affordability of healthcare services have been more comprehensively studied than other aspects of access. These dimensions are more readily quantified from publicly available health care workforce and insurance status data. Availability of services has decreased because of a surge in rural hospital closures in the past ten years, which has disproportionately affected rural minority populations [59]. Similarly, rural minority populations, particularly American Indian/Alaska Native populations, tend to live farther from healthcare services (i.e., less accessibility), including cancer care services such as National Cancer Institute (NCI)-designated cancer centers and cancer care specialists [60,61]. Affordability is affected by the inability of rural minority populations to access health insurance, either as a result of limited Medicaid expansion in states with large rural minority populations or because of the Affordable Care Act regulations associated with immigration status [62]. Furthermore, compared to their White counterparts, racial/ethnic minority populations are more likely to face financial hardship in paying for cancer care [63].

Acceptability is influenced by both racial/ethnic minority populations’ expectations of, and reality with, experiencing discrimination in healthcare systems and their mistrust of healthcare providers as a result of historical unethical and criminal behaviors, such as unauthorized treatments and experiments performed on Black people, and present-day biases (e.g., pain perception) [64,65,66]. Acceptability may be facilitated by racial–ethnic concordance between patients and providers [67]. However, the closure of many Black medical schools in the 20th century may have reduced the number of Black physicians by ~35,000 roughly over the last century. In 2019, only 6.2% of medical school graduates were Black, less than half the proportion of the Black population nationwide [68]. This is particularly concerning in rural areas with large minority populations, as these areas already have a shortage of physicians.

Relatedly, racial/ethnic minority populations may face stigma accessing key healthcare infrastructure, which led the American College of Physicians to issue a recent call to reduce such inequities [69]. Lack of accommodation in healthcare access may include limited hours of clinic operation or lack of translation services. These limited hours may have a disproportionate effect on rural and racial/ethnic minority populations who may be more likely to work for small businesses with less flexible work-leave policies or who are more likely to work in manufacturing and service professions for which there are multiple shifts [70,71]. Furthermore, rural racial/ethnic minority persons whose primary language is not English may face additional challenges with language barriers in healthcare settings, which is especially critical with the growing population of immigrants from Spanish-speaking countries who now reside in rural America [14].

### 3.3. Socioeconomic Status, Educational Attainment, Housing, and Infrastructure

**Figure 3:**
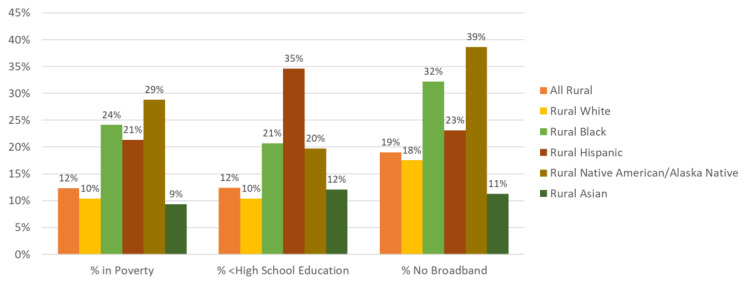
Social determinants of health across rural racial/ethnic groups (findings reported by Probst et al. based on American Community Survey data) [77,78,79,80].

Generally, rural populations have higher rates of poverty, lower educational attainment, and more limited or aging community infrastructure than their urban counterparts [28,72]. Racial/ethnic minority populations, especially those living in rural areas, are disproportionately affected by these social and structural factors [73]. Historical racism and its long-term effects may play a role in these contextual factors [74]. Residential segregation also has led to concentrated Black poverty, as is seen by the clusters of persistent poverty along the Lower Mississippi River [74,75]. Furthermore, the impact of historical racism has had long-term effects on educational attainment with segregation shown to be associated with high school and college graduation among Black students, but not White students [74,76]. Poverty and educational attainment are both worse among rural racial/ethnic minority residents in comparison to their White counterparts. Poverty is higher among rural American Indian/Alaska Native (29%), non-Hispanic Black (24%), and Hispanic (21%) residents compared to White (10%) and Asian (9%) rural persons (Figure 3) [77,78,79,80]. Among rural residents, the highest rates of those with less than a high school diploma were among Hispanic (35%), Black (21%), and American Indian/Alaska Native (19.7%) populations compared to White (10%) and Asian (12%) populations [77,78,79,80]. Major housing inadequacies, such as plumbing issues, occur in a higher proportion of rural areas and decrease as areas become more metropolitan. Infrastructure issues, including lower access to safe roads, public transportation, and broadband, also exist in rural areas [77,78,79,80,81]. These social and structural determinants contribute to disparities in cancer outcomes among rural and racial/ethnic minority populations by inducing persistent stress, enabling or promoting poorer health behaviors, reducing access to cancer care, and affecting guideline-concordant treatment and cancer survivorship [73,82,83,84,85,86].

### 3.4. Environmental Factors

Rural areas, particularly those with a higher proportion of residents reliant on well water and agricultural or mining industries, may be at an increased risk of exposure to carcinogens such as arsenic, radon, and nitrate [87]. Rural populations may be at greater risk of exposure to radon because of lower rates of home testing and, therefore, subsequent mitigation [88]. However, many of the previous studies that focused on well water, agricultural exposures, and radon testing and mitigation have been concentrated in the Northeast and Midwest, where rural populations are largely White [89,90,91]. Historical and contemporary environmental racism may affect rural, racial/ethnic minorities’ exposures to carcinogens. Examples include the placement of landfills for carcinogenic polychlorinated biphenyl (PCB) in rural Warren County North Carolina in 1982, petrochemical exposures in “Cancer Alley” in rural, largely Black Louisiana, and exposures to carcinogens among tribal populations due to mining near reservations [91]. “Cancer Alley” is a 100-mile stretch between Baton Rouge and New Orleans, Louisiana, that includes the largely rural St. John the Baptist and St. James’ counties. One-quarter of the nation’s petrochemical production occurs in this area and includes a population that is 40% Black [92]. These kinds of exposures can lead to a greater risk of cancer incidence and poorer cancer outcomes. For example, recent studies from North Carolina showed that land quality, as indicated by agricultural activity, pesticide usage, and toxic release and priority cleanup sites, was associated with later stage at diagnosis for breast cancer among rural women [93]. Identifying the independent causal association between environmental exposures and cancer incidence is difficult at a population level, but it is critical to understanding and mitigating rural and racial/ethnic disparities in cancer [94,95].

## 4. Rural and Racial/Ethnic Disparities in Cancer Across the Continuum

### 4.1. Risk Factors and Primary Prevention

Significant differences in health behaviors have been observed across rural–urban and racial/ethnic subgroups, including tobacco use, alcohol consumption, diet, physical activity, and human papillomavirus (HPV) vaccination. Rural populations tend to have higher rates of tobacco use, diets higher in red/processed meat and lower in fruit/vegetable consumption, lower levels of physical activity, and lower uptake of HPV vaccination, but also tend to have lower alcohol consumption compared to urban areas [2,3,4]. Cancer-relevant health behaviors vary uniquely by race/ethnicity (e.g., smoking is higher among American Indian/Alaska Native populations, but Black populations have lower rates of physical activity) [55].

Smoking and smokeless tobacco use vary among rural racial/ethnic minority groups. Historically, American Indians and Alaskan Natives have been identified in national surveys, such as the Behavioral Risk Factor Surveillance System (BRFSS), as having the highest rates of current smoking among rural persons (36.7%), with rates higher than those seen in other racial/ethnic minority groups: non-Hispanic Black (23.2%), Hispanic (17.0%), and Asian/Pacific Islander/Native Hawaiians (10.9%) [55]. Studies have also shown intrarural differences in smoking among American Indian/Alaska Native populations, with those living on tribal lands having lower current smoking prevalence compared to those living in non-tribal rural areas [96]. There are some regional and tribal variations in smoking prevalence among American India/Alaska Native populations, even within a specific state. For example, smoking prevalence among rural Alaska Natives varies notably across regions of the state [97]. It is important to note, however, that smoking in ceremonial settings is an important part of many tribal cultures [98]. This may affect the interpretation of national studies and the representation of smoking prevalence among these populations. Additionally, national surveys have shown that smoking rates vary by rurality and racial/ethnicity, e.g., among rural Black persons, smoking rates are lower than among urban Black persons [99]. Smokeless tobacco use tends to be most prevalent among rural White populations when a dichotomous analysis of race/ethnicity is considered (i.e., White vs. non-White) [100]. However, studies have shown that rural Alaskans, particularly Native Alaskans, have a higher prevalence of smokeless tobacco use compared to their White rural counterparts [101]. A rural North Carolina study showed that Black and Native American persons were more likely to use “snuff”, whereas Native Americans were more likely to use chewing tobacco compared to their White counterparts [102]. Another study showed that among a rural cohort of adolescents in California, non-Hispanic White teens were more likely to use smokeless tobacco compared to their rural Hispanic counterparts [103].

Alcohol use, which is associated with multiple cancer types, including forms of the liver, mouth, and esophagus, differs across rural–urban and racial/ethnic minority populations [104]. Binge drinking rates are lower among rural Black and Asian/Pacific Islander populations compared to rural White adults, but there was no difference between White adults and American Indian/Alaska Native or Hispanic adults [55]. Other recent studies focused on adolescent alcohol use have yielded similar findings [105]. Within the rural Hispanic farmworker population in North Carolina, the risk of alcohol abuse was found to be positively associated with years of residence in the United States [106]. This finding underscores the importance of considering the role of assimilation in cancer risk among rural Hispanic populations, as some rural areas are home to both newly immigrated and long-residing Hispanic populations.

Diet is a complex cancer-relevant health behavior that, depending on the foods consumed, can either decrease (e.g., fruits and vegetables) or increase (e.g., red and processed meats) risk for developing cancer [107]. A comprehensive study of consumption habits is critical in rural communities, given the strong overlap between food deserts and rural areas [108,109]. This intersection of rurality and diet is directly visible among different racial/ethnic groups. For example, in the Navajo Nation, 57% of residents in the Community Outreach and Patient Empowerment Program study stated they did not consume enough fruits and vegetables. Of these individuals, 61% cited expense as a reason, and over 51% reported needing to travel over an hour to obtain most of their food (which may have discouraged the purchase of fresh, perishable items) [110]. Similarly, a study in rural North Carolina found that rural Black older adults had a better Total Health Eating Index-2005 score compared to their White and American Indian counterparts, but the groups varied in their consumption of different types of healthy foods. However, no group had an overall high-quality diet [111]. Studies based in other states (Mississippi) have shown that rural Black persons have poorer diets than their rural White counterparts and the nation as a whole, suggesting that there may be geographic variation in these disparities [112]. The influence of these cultural and geographic-related factors on healthy diets among rural racial/ethnic minority groups demonstrates the need for tailored interventions for different populations to address the cancer burden, paying particular attention to the traditional food choices of particular regions and/or cultures.

In a similar vein, there is a connection between high body mass index and lack of physical activity and many types of cancer (e.g., colorectal cancer, postmenopausal breast cancer, pancreatic cancer). Studies have shown that rural populations have higher rates of obesity and lower levels of physical activity compared to their urban counterparts and that obesity/physical activity varies by race/ethnicity among rural populations [3,55]. Among rural populations, Black, Hispanic, and American Indian/Alaska Native populations had higher rates of obesity than their rural White peers, although roughly a third or more of all these groups were obese (ranging from 32.0% among rural White adults to 45.9% of rural Black adults) [55]. Rural Asian/Native Hawaiian/Other Pacific Islander populations had the lowest rate of obesity (15.5%) [55]. Other studies have shown that rates of obesity are higher among rural Black and White populations compared to their respective urban counterparts [113]. These higher rates of both obesity and physical inactivity may be due, in part, to obesogenic built environments that may be more prevalent in areas with larger racial/ethnic minority populations (i.e., the South) [114,115].

In addition to health behaviors that may put rural racial/ethnic minority populations at greater risk for cancer, it is important to assess the prevalence of health-promoting behaviors such as HPV vaccination, which can protect against several cancer types (e.g., cervical cancer, oropharyngeal cancer) [116]. Both rural and Black populations have higher incidence and mortality rates of these preventable, HPV-associated cancers [5,117]. HPV vaccination initiation rates are consistently lower among rural adolescents overall and among rural White and Hispanic adolescents specifically [118]. There has been no statistically significant difference in HPV vaccination initiation among Black adolescents across rural–urban designations [118]. Another recent study showed that vaccination rates were higher among Black and Hispanic teens compared to their White counterparts regardless of rural–urban residence [4]. Although vaccination rates have been more favorable among rural racial/ethnic minority populations, studies have shown that lower knowledge of the role of HPV vaccination in cancer prevention and financial and psychosocial barriers may play a role in lower rates of HPV vaccination in rural areas and among some rural racial/ethnic minority populations, such as newly immigrated Hispanics [119,120].

Racial segregation may play a role in many of these health behaviors that are more common among rural racial/ethnic minority populations. For example, studies show that Black persons in more segregated areas have higher smoking rates and have less access to healthy foods [121,122]. However, these types of studies examining the relationship between residential segregation and cancer-related health behaviors have primarily been performed in urban areas. Examining rural residential segregation is an important area of needed research to help explain and address cancer-related health behaviors [123].

### 4.2. Cancer Screening

For many cancer types (e.g., breast, colorectal, cervical, lung), receipt of screening at the recommended ages and intervals can detect cancer at an earlier, more treatable stage, improving outcomes and survival. Lower utilization of screening services in rural areas and areas with less geographic access to screening services have been well documented [124,125,126]. However, there have been mixed findings on racial/ethnic differences overall and at the intersection of race/ethnicity and rurality. Some studies have found that in rural communities, ethnicity (Hispanic vs. non-Hispanic) was not associated with having a guideline-concordant mammogram [127]. Disparities in race were seen in other studies that showed that rural Hispanic women were less likely to have had a mammogram within the past year compared with urban Hispanic women [128]. Rural Black women in persistent-poverty counties were less likely to report a recent mammogram compared to their urban counterparts [125]. However, another study found that for breast and cervical cancers, Black women had favorable odds of receipt of service regardless of rurality [129]. Multiple studies have shown that rural American Indian populations have lower rates of cancer screening compared to their urban counterparts, with lower rates found among those living in the most remote areas [128,130]. Rural Black persons had a lower probability of reporting colorectal cancer screening than urban Black persons (44.8% vs. 51.8%) [7]. Similarly, rural Hispanics/Latinos had a lower predicted probability of reporting colorectal cancer screening than urban Hispanics/Latinos (40.8% vs. 43.7%) [7]. Since its recommendation by the United States Preventive Services Task Force (USPSTF) in 2013, lung cancer screening uptake at the intersection of race/ethnicity and rurality has been difficult to assess because of limited data availability and small sample sizes [131,132]. However, initial analyses of national surveys have shown that there are no rural–urban or racial/ethnic differences in screening uptake [132,133,134]. Studies have shown that lack of recommendation from a provider is a key barrier to being up to date with screening among rural patients [135]. Furthermore, low quality of provider information has been identified as a barrier to screening among rural Black individuals [136]. Provider biases may play a role in lack of provider recommendations and/or low-quality recommendations. Further research is needed to examine the role of biases in cancer screening recommendations and referrals among rural, racial/ethnic minority patients.

### 4.3. Cancer Incidence and Staging

We provide a summary of age-adjusted incidence rates for all cancers combined and several common cancer types across rural/urban designations and racial/ethnic groups for the most recent 5-year period of available data (2013–2017) using SEER 21 data (Table 1). 

Generally speaking, rural, racial/ethnic minority populations have had higher incidence rates for lung and colorectal cancers. Rural Black populations had the highest rates of colorectal, female breast, prostate, and cervical cancer across rural groups. Previous studies utilizing more representative national data have shown that overall cancer incidence is higher among urban populations, driven in large part by higher rates of breast and prostate cancer, for which access to and utilization of screening may be a driving factor [5,6]. This holds across racial strata, except for American Indian/Alaska Native populations, among whom the all-cancer incidence rate was higher among rural populations, and Asian/Pacific Islanders, among whom there was no rural–urban difference [6]. However, for cancers with modifiable risk factors (e.g., tobacco-associated and HPV-associated cancers) or preventive screening opportunities (e.g., colorectal cancer), incidence rates are higher in rural compared to urban populations, with greater disparities among rural non-Hispanic Black persons, although rural Hispanic persons tended to experience lower incidence compared to their urban peers [5,6]. Early-onset colorectal cancer (i.e., colorectal cancers in those aged 20–49) has increased in rural populations since 2000, with persistently high rates among rural Black populations, and with the highest recent rate seen among rural American Indian/Alaska Native populations [138]. For HPV-associated cancers broadly and cervical cancer specifically, rates are highest for rural Black persons [5]. For cervical cancer, rural Black, White, and American Indian women have the higher rates compared to their urban counterparts [139]. Black men tended to have higher lung cancer incidence rates across all levels of rurality and for all histology types except small cell [140]. Although urban populations tend to have higher rates of testicular cancer overall, rural American Indian/Alaska Native and Asian/Pacific Islanders have higher rates compared to their urban counterparts, whereas other racial/ethnic groups experienced no rural–urban differences or showed more favorable rates in rural populations [141]. Studies have also tended to show that rural populations, regardless of race/ethnicity, are diagnosed with cancer at a more advanced stage, particularly for cancers with primary and secondary preventive opportunities, such as lung, cervical, and colorectal cancers [142,143].

### 4.4. Cancer Treatment

Although studies have shown that rural and racial/ethnic minorities are less likely to receive certain cancer treatments or receive them in accordance with guidelines, few studies have examined the intersection of rurality and race/ethnicity in their effects on cancer treatment [144,145]. Those studies that have explored this intersection have identified notable disparities among rural racial/ethnic minority cancer patients. For example, in the treatment of early-stage non-small cell lung cancer (NSCLC), complete surgical resection is considered the best treatment option to increase survival. However, in an analysis of 3481 Alabama Medicare beneficiaries diagnosed with NSCLC, the proportion of rural Black patients (10.7%) receiving surgery at any point was significantly less than the proportion of rural White patients (28.7%) [146]. A study examining surgical treatment for endometrial cancer found that the proportion of Black women who did not undergo a lymphadenectomy, a procedure to remove and examine lymph nodes to help determine cancer progression, was significantly higher among those in rural compared to urban areas (40.9% vs. 30.3%) [147]. Multivariable analysis of national disparities in laparoscopic procedures for colon cancer treatment found similar results: the odds of undergoing less-invasive laparoscopic procedures were significantly higher among Black (OR: 2.2; 95% CI: 1.9, 2.5) and Hispanic (OR: 2.2; 95% CI: 1.9, 2.5) patients that were cared for in urban areas compared to their rural counterparts [148]. Several other studies have demonstrated that, when examined independently, racial/ethnic minorities and persons living in rural or non-metro areas have lower proportions and/or odds of receiving higher-quality cancer treatment across multiple modalities for a variety of cancers [149,150,151,152,153,154]. Furthermore, studies have shown that rural Black and Hispanic cancer patients were less likely to report easily getting care compared to White patients [155]. Some studies, although not specifically evaluating disparities in treatment, provide insight into why certain racial/ethnic disparities are likely to be present among rural populations. In a study of breast cancer patients from multiple U.S. states, Black (OR=1.93; 95% CI: 1.17, 3.18) and Hispanic (OR= 2.01; 95% CI: 1.21, 3.35) women were found to have significantly higher odds of being treated at low-volume hospitals [156]. Low-volume hospitals are much more prevalent in rural areas and, according to some studies, have been shown to offer lower-quality care in comparison to higher-volume hospitals [157,158].

Rural, racial/ethnic minority cancer patients are less likely to receive guideline-concordant care for their cancer, particularly when it comes to cancer surgery. This may be due in part to these populations being more frequently treated at low-volume hospitals. More research is needed to examine cancer treatment disparities across cancer sites and treatment modalities and to determine the independent contributions of contextual and treatment location factors. Furthermore, it is imperative to understand the dynamics between patient preference, provider biases, and provider–patient communication [159]. Studies have shown that perceived racism, distrust of the healthcare system, and providers’ spurious perceptions of treatment efficacy play a role in the receipt of cancer treatment [159,160,161,162]. However, studies have yet to examine if and to what magnitude these factors may play a role in the treatment disparities experienced by rural racial/ethnic minority patients.

### 4.5. Cancer Survivorship

Survivorship comprises the health and wellbeing of a person with cancer from diagnosis until the end of life, including physical, mental, social, emotional, and financial effects of cancer that start at diagnosis and continue beyond treatment [163]. Rural and racial/ethnic disparities in survivorship issues (e.g., long-term follow-up care/surveillance, quality of life, financial toxicity) have been identified [164,165,166,167,168,169]. Rural cancer survivors have the highest rates of poor self-reported health, physical distress, and activity limitations as compared to their urban counterparts, as well as reported lower receipt of guidance about cancer follow-up care [167]. In addition, rural cancer survivors had poorer mental health outcomes compared to urban cancer survivors [165,168]. Studies have shown that rural cancer survivors report higher likelihood of financial toxicity compared to their urban counterparts, but this association is attenuated upon adjustment for other factors [170,171]. Studies have also identified cancer survivorship disparities among racial/ethnic minorities. Black and Hispanic breast cancer survivors were less likely to have surveillance mammography compared to their White counterparts. Other studies have shown that Black and Hispanic cancer survivors were more likely to forego prescription medications and dental care because of cost or experienced greater material and psychological financial hardship than White cancer survivors [172,173]. However, the intersection of rurality and race/ethnicity and their effect on cancer survivorship has been only minimally explored. A study among breast cancer survivors in the Carolina Breast Cancer Study found that rural survivors were most financially affected by their diagnosis, but the magnitude of this impact was greatest among rural Black survivors [174]. Another study in South Carolina found that Black breast cancer survivors, particularly those in rural areas, had lower medication possession ratios, indicating greater nonadherence to endocrine therapy [85]. Opportunities exist to further quantify the intersection of race/ethnicity, rurality, and cancer survivorship, particularly among understudied American Indian/Alaska Native populations and the role that palliative care plays in cancer survivorship [175].

### 4.6. Cancer Mortality and Survival

In Table 2, we provide cancer mortality rates across racial/ethnic groups in both rural and urban areas. 

Rural Black populations have the highest cancer mortality rate, and cancer mortality rates among rural White and Black populations exceed that of their urban counterparts. Lung cancer mortality rates are higher among all rural racial/ethnic groups compared to urban. For colorectal and prostate cancer, rural Black populations have the highest mortality rate with rural White, Black, and American Indian/Alaska Native populations having higher mortality rates than their urban peers. Rural racial/ethnic disparities in cervical cancer mortality exist for all groups except Asian/Pacific Islander. Past studies have consistently shown that rural racial/ethnic minority patients have the poorest cancer-specific survival and mortality rates. Among all population groups, the rural Black population has the highest cancer mortality rate, but rural American Indian/Alaska Native populations have the largest disparity compared to their urban counterparts (a 33% higher rate) [41]. Studies using multiple categories of rurality have found that White, Black, and American Indian/Alaska Native populations have the highest cancer mortality burden [177,178]. Furthermore, despite overall improvement in cancer mortality trends, low-income Black patients in non-metropolitan areas experienced two to three times higher premature mortality risks than affluent Black and White patients in metropolitan areas. These associations were true for specific cancers as well. Studies have reported that living in rural areas increased the risk of mortality from lung cancer for Black patients overall by 54% and for Black women by 29% [179,180]. In colorectal cancer, Higginbotham et al. found that rural Black women had a 30% higher mortality rate than their urban counterparts, although Hines and colleagues found that the interaction term of Black race and rural residence was not statistically significant in models [180,181]. For breast cancer, one study found that in the urban setting of their study (Chicago communities), 17 out of 20 communities with the highest breast cancer mortality rates were predominantly Black, and that in the rural setting of their study (rural Washington state), Hispanic women had higher breast cancer mortality compared to non-Hispanic White women [182]. For cervical cancer, two studies found that Black women had higher mortality rates than White women regardless of geography, despite overall improvements among rural and urban women [139,183]. However, prostate cancer mortality rates in non-metropolitan areas compared to metropolitan areas were 12% higher in Black men and only 4% higher in White men [184]. Finally, another study provided results showing that urban American Indian and Alaska Native patients had a significantly higher risk of all-cancer mortality than urban White patients [185]. In children and adolescents in the state of Tennessee, Lindley and colleagues found that rural areas were more likely to be cancer mortality clusters and that the odds of living near a mortality cluster were nearly three times as high for Black children and adolescents (OR: 2.92, 95% CI: 1.13, 7.54) as their White counterparts [186].

To a lesser extent, cancer survival has been explored as a cancer outcome at the intersection of rurality and race/ethnicity, although some studies have examined rurality and race/ethnicity independently [147,187]. Generally, rural Black and American Indian/Alaska Native populations had the poorest survival outcomes. Five-year survival rates for cervical cancer among Black women in rural areas were 10% lower than Black women in metropolitan areas and 20% lower than White women in metropolitan areas [139]. In children and adolescents, American Indians/Alaska Natives residing in non-metro areas had more than twice the increased risk of cancer death than White residents in metro areas [188]. An analysis of National Cancer Database data showed that rural Black head and neck cancer patients had the shortest median survival time (35.1 months compared to 67 months among White urban patients) [189]. In rural–urban stratified analyses, Black men had poorer colorectal cancer survival than their White counterparts, with the greatest disparity among rural men; rural Asian men also had poorer survival outcomes [190].

## 5. Implications/Recommendations

We examined the extant research on cancer disparities among rural and racial/ethnic minority populations, extending from the social determinants of health that affect cancer disparities across the continuum. We summarize key findings from observational studies in the literature that explored disparities in primary prevention activities through survivorship among rural racial/ethnic minority populations. Our review notes several key opportunities for further policy changes and new areas for observational and interventional research to improve these identified disparities. Policy changes are critical to ensure that physical, social, and healthcare environments are optimized for health equity, particularly for those rural racial/ethnic minority populations that experience that greatest cancer burden: Black and American Indian/Alaska Native populations. Although we summarize many studies that have characterized the disparities at the intersection of rurality and race/ethnicity, continued research is needed to further elucidate and determine the appropriate actions to address these disparities. Furthermore, more studies are needed among American Indian/Alaska Native populations to address the particular burden among those individuals, and with the changes in availability and updated USPSTF recommendations, more research is needed to address disparities in lung and colorectal cancer among Black populations. Finally, there is a need for evidence-based interventions to be tested, implemented, and adopted in a culturally competent manner to improve cancer prevention and control activities at the community and clinic levels in rural settings.

To improve the social contextual and healthcare factors that affect cancer disparities across the continuum for rural racial/ethnic minority populations, it is important to develop and implement policies—at all levels from the federal government to local healthcare systems—that not only reduce disparities and biases but also promote equity. Our review notes that centuries of discriminatory policies (e.g., Jim Crow, redlining) have long-lasting effects on cancer outcomes that disproportionately affect racial/ethnic minority groups and that provider biases may affect screening and treatment disparities. It is critical to continue to examine the effects of these policies and biases long-term but also to implement policies that promote equity, including ensuring diversity in the healthcare workforce, enhancing community partnerships, and addressing institutional racism, as supported by the American Society of Clinical Oncology [191]. Diversity in the rural cancer care workforce, in particular, may be an effective means of addressing these disparities. Historically Black Colleges and Universities and student pipeline programs can play a key role in educating Black students and future healthcare professionals (e.g., Morehouse College’s IMHOTEP internship program) [192]. In August 2020, Oklahoma State University and the Cherokee Nation opened an osteopathic medical school on Cherokee tribal lands with a goal of training future physicians, particularly those who are American Indian, to later practice in rural areas [193]. Improving representation among racial/ethnic minorities may help reduce provider biases and improve cancer care.

It is also important to consider the role of region in modifying the relationships observed between rurality, race/ethnicity, and cancer outcomes. The stark differences in regions’ demographic and sociopolitical environments and physical access to healthcare-related services necessitates that researchers aim to determine whether observed rural racial/ethnic disparities hold uniformly across the U.S. or exist only in particular regions/localities, thereby requiring a different policy approach. Many states with high rural racial/ethnic minority populations in the South have not expanded access to Medicaid, which would afford many low-income Black and Hispanic populations greater access to primary and oncology care and has been shown to improve the use of cancer prevention services and cancer outcomes [194,195,196]. Much of the existing research on rural cancer disparities has focused on Appalachia, which experiences significant cancer burden; however, the Mississippi Delta and Black Belt regions of the country experience an even greater cancer mortality burden, as well as lower rates of cancer-prevention behaviors (e.g., HPV vaccination) and reduced access to care [117,197,198,199]. Much like Appalachia, the Delta Region (i.e., the Delta Regional Authority) is a federally designated region for socioeconomic development and receives federal funding for economic development and some health programming as well, as it is a designated area for loan repayment programs and visa waivers to increase access to healthcare providers. However, it is under-resourced, with the fiscal year 2020 budget allocating 45 cents for every dollar per capita directly to the Delta Regional Authority compared to the Appalachian Regional Commission (not including regionally targeted funding from other federal agencies) [200,201]. More resources need to be targeted to this region, which has a high proportion of rural Black residents who experience less access to care and greater cancer burden.

Although a growing number of studies are exploring urban–rural disparities in health behaviors and outcomes across the cancer continuum and adding to the larger existing racial/ethnic disparities literature, fewer studies have focused on the intersection of race/ethnicity and rurality. To overcome this gap, there is first a collective need for increased awareness of the compounding effects of racial/ethnic minority status and rural residence in the U.S. Second, the effect of data collection and privacy rules on researchers’ ability to study this important issue must be considered. Many population-based datasets have placed restrictions or moratoriums on accessing geographic-specific identifiers, including urban–rural status; however, in recent years, widely used datasets such as the National Health Interview Survey and BRFSS have begun incorporating such variables into their public-use files with minimal missingness. Unfortunately, many datasets are only available to access at federal Research Data Centers (RDCs), with costly data application/analysis fees, and some data remain masked because of privacy concerns (e.g., Medical Expenditures Panel Survey, Medicare data) [202,203]. Finally, because of administrative constraints and sampling approaches used in many population-based surveys, it is difficult to reach the sample sizes necessary to look at combinations of social strata (i.e., racial/ethnicity by rurality). Addressing this issue will likely require additional funding and/or oversampling to ensure both representative samples and large enough subgroup samples are obtained to make comparisons. The progress of observational research at the intersection of race/ethnicity and rurality hinges on dealing with these challenges.

As shown from our narrative review above, more research is needed on our smallest population groups, particularly rural American Indian/Alaska Natives, who experience a high cancer burden. As a result of small sample sizes, restrictive access to Indian Health Services data files, and insufficient payor categories (e.g., public vs. private), researchers know very little about whether American Indian/Alaska Native populations are receiving timely and high-quality care across the cancer continuum. Similarly, the challenge of small rural sample sizes in population-based surveys and other national data is exacerbated when rural American Indian/Alaska Natives are considered [204]. This is a particularly critical issue, as multiple studies have shown that American Indian/Alaska Native populations have the farthest travel burden to many cancer specialists and NCI-designated cancer centers and have the greatest rural–urban cancer mortality of any racial/ethnic group in the U.S. [41,60,61].

Furthermore, as new screening, diagnostic, treatment approaches emerge, it is important to understand how access to and utilization of these services vary based on rurality and race/ethnicity. For example, low-dose CT screening for lung cancer was initially recommended by the USPSTF in 2013, but critics were concerned that the high pack-year history requirements meant that fewer Black smokers may be eligible for screening [205,206]. Subsequently, the USPSTF released a draft statement that, if implemented, would lower the screening age and pack-year eligibility requirement [207]. Thus, this change may enable more rural Black residents, who experience a notable lung cancer burden, to be eligible for screening [140]. Although early national, population-based analyses of lung cancer screening utilization have not identified rural and/or racial disparities in screening, this is an important area for future research [132,134]. As our review notes, some research has examined disparities in the receipt and quality of cancer surgery, chemotherapy, and radiation treatments for cancer. In recent years, costly treatments such as immunotherapies and targeted therapies have been increasingly prescribed and are thus an important area for disparities research to ensure that innovations increase equity rather than intensify disparities [208,209,210].

Recent reports by the U.S. Department of Health and Human Services have outlined the critical need to focus research activities in rural communities to address disparities in the form of a rural health action plan and the need for social and behavioral intervention research [211]. The historical scarcity of federally funded cancer research in rural settings has led to an increased focus on cancer prevention in these settings, especially in light of documented disparities in cancer outcomes [212,213,214,215]. There has been a limited focus on cancer prevention and control intervention research in rural settings [216]. When examining NCI’s Evidence-Based Cancer Control Programs, only 40 (~25%) of 202 evidence-based interventions reported the community type as including rural with other settings, such as rural with suburban and urban/inner-city settings [217]. Of those 40, only 13 focused exclusively on rural communities. It is unclear how many of these evidence-based interventions in rural settings encompassed a focus on the intersection of rural life with myriad other factors. There is a need for clear guidance on developing interventions to accommodate the multiple and simultaneous forms of identity in rural communities. One without the other results in insufficient context to inform adaptation from one rural setting to the next and likely ineffective interventions on cancer-related outcomes.

Moreover, existing reporting guidelines aimed at promoting consistency in intervention reporting and replication and translating evidence into practice could promote more detailed geographic descriptions in their requirements to better understand rural context and intersectional identity among participants. Across existing frameworks and applications, the need to address intersectional identity matters. The AIMD framework considers the Aims, Ingredients, Mechanism, and Delivery of an intervention, which could provide context about the rural setting, rural-residing populations, and logistical and practical considerations of working in rural communities [218]. Similarly, the Standards for Reporting Implementation Studies (StaRI) checklist includes the opportunity to describe the context and targeted sites of an intervention and any contextual changes that may have affected the intervention’s outcomes [219]. The Template for Intervention Description and Replication (TIDieR) also includes a focus on where the intervention occurred, including infrastructure or other relevant features [220]. Lastly, the equity extension to Consolidated Standards of Reporting Trials (CONSORT) calls for reporting who the study participants are, plus their relationship with their setting, given the interconnectedness of person and place, as well as the context and relationship of the study settings and locations to health inequity and generalizability [221]. The CONSORT equity extension demonstrates a promising approach to fully describing and understanding the importance of intersectional identity, especially in rural settings.

## 6. Conclusions

We provide a comprehensive review of the geographic distribution of rural racial/ethnic populations, provide a framework for and describe how the social determinants of health may disproportionately increase the risk of cancer disparities in these populations across the continuum, and detail a summary of the disparities these populations experience from cancer-relevant preventive behaviors to mortality. We identified that rural Black and American Indian/Alaska Native populations often experience the greatest cancer burden. As such, policies, as well as observational, interventional, and implementation research, must address the intersection of rurality and race/ethnicity to ensure that disparities are reduced, and equity is promoted.

## Figures and Tables

**Table 1:** Rural and urban age-adjusted cancer incidence rates across racial/ethnic groups, 2013– 2017.

Racial/Ethnic Group	Age-Adjusted Rural Incidence Rate per 100,000	Age-Adjusted Urban Incidence Rate per 100,000
**All Cancers**		
Non-Hispanic White	471.9	470.6
Non-Hispanic Black	455.9	454.1
American Indian/Alaska Native	338.5	337.8
Asian/Pacific Islander	320.3 *	305.9
Hispanic	322.9	349.3 *
**Lung Cancer**		
Non-Hispanic White	71.6 *	58.5
Non-Hispanic Black	68.1 *	56.1
American Indian/Alaska Native	35.7	41.2 *
Asian/Pacific Islander	40.5 *	36.4
Hispanic	31.3 *	28.9
	**Colorectal Cancer**	
Non-Hispanic White	43.6 *	37.7
Non-Hispanic Black	52.7 *	44.6
American Indian/Alaska Native	42.3 *	33.1
Asian/Pacific Islander	34.8	32.2
Hispanic	35.3	33.7
**Prostate Cancer**		
Non-Hispanic White	99.7	106.0 *
Non-Hispanic Black	166.7	181.9 *
American Indian/Alaska Native	64.3	64.0
Asian/Pacific Islander	61.2	57.2
Hispanic	69.6	93.0 *
**Female Breast Cancer**		
Non-Hispanic White	120.1	139.7 *
Non-Hispanic Black	124.2	128.9
American Indian/Alaska Native	80.4	94.1 *
Asian/Pacific Islander	109.4	104.3
Hispanic	89.7	99.4 *
**Cervical Cancer**		
Non-Hispanic White	8.6 *	6.5
Non-Hispanic Black	10.8 *	8.8
American Indian/Alaska Native	10.8	7.9
Asian/Pacific Islander	6.6	6.6
Hispanic	8.4	9.2

Note: Rates are age-adjusted per the 2000 U.S. Standard Population and calculated from the Surveillance Epidemiology and End Results (SEER) 21 April 2020 data release [137]. Data from the Alaska and Hawaii Registries are not included as rural–urban indicators are not available. * indicates a statistically significant higher rate among a racial/ethnic group compared to their geographic counterpart.

**Table 2:** Rural and urban age-adjusted cancer mortality rates across racial/ethnic groups, 2013–2017.

Racial/Ethnic Group	Age-Adjusted Rural Mortality Rate per 100,000	Age-Adjusted Urban Mortality Rate per 100,000
**All Cancers**		
Non-Hispanic White	176.0 *	160.1
Non-Hispanic Black	199.1 *	184.4
American Indian/Alaska Native	161.2 *	120.8
Asian/Pacific Islander	104.7	98.4
Hispanic	109.1	111.0
**Lung Cancer**		
Non-Hispanic White	50.0 *	41.8
Non-Hispanic Black	49.2 *	42.6
American Indian/Alaska Native	40.0 *	29.8
Asian/Pacific Islander	24.6 *	22.0
Hispanic	48.5 *	38.4
	**Colorectal Cancer**	
Non-Hispanic White	15.8 *	13.3
Non-Hispanic Black	22.0 *	18.5
American Indian/Alaska Native	18.6 *	11.8
Asian/Pacific Islander	9.9	9.5
Hispanic	16.0 *	13.4
**Prostate Cancer**		
Non-Hispanic White	18.6 *	17.8
Non-Hispanic Black	40.7 *	37.9
American Indian/Alaska Native	20.7 *	14.2
Asian/Pacific Islander	8.9	8.5
Hispanic	13.0	15.7 *
**Female Breast Cancer**		
Non-Hispanic White	20.5	20.4
Non-Hispanic Black	28.6	28.5
American Indian/Alaska Native	15.9	14.2
Asian/Pacific Islander	12.5	11.4
Hispanic	20.7	20.3
**Cervical Cancer**		
Non-Hispanic White	2.5 *	2.0
Non-Hispanic Black	4.6 *	3.4
American Indian/Alaska Native	3.4 *	1.9
Asian/Pacific Islander	1.8	1.8
Hispanic	2.7 *	2.2

Note: Rates are age-adjusted per the 2000 U.S. Standard Population and calculated from the CDC Wonder Dataset [176]. * indicates a statistically significant higher rate among a racial/ethnic group compared to their geographic counterpart.

## Data Availability

Publicly available datasets were analyzed in this study. These data sources are cited in the footnotes below Table 1 and Table 2 and in a corresponding position within the references.
